# SGLT‐2 Inhibitors in Heart Failure: Current Management, Unmet Needs, and Therapeutic Prospects

**DOI:** 10.1161/JAHA.119.013389

**Published:** 2019-10-12

**Authors:** Carolyn S. P. Lam, Chanchal Chandramouli, Vineeta Ahooja, Subodh Verma

**Affiliations:** ^1^ National Heart Centre Singapore Singapore; ^2^ Duke‐National University of Singapore Medical School Singapore; ^3^ University Medical Centre Groningen Groningen the Netherlands; ^4^ The George Institute for Global Health Newtown Australia; ^5^ Heart Health Institute Toronto Ontario Canada; ^6^ Division of Cardiac Surgery Keenan Research Centre for Biomedical Science and Li Ka Shing Knowledge Institute of St Michael's Hospital University of Toronto Toronto Ontario Canada

**Keywords:** heart failure, SGLT‐2 inhibitors, type 2 diabetes mellitus, unmet needs, Heart Failure, Diabetes, Type 2, Cardiorenal Syndrome

## Introduction

Heart failure (HF) is a growing public health issue. As many as 1 in 5 people are expected to develop HF during their lifetime,^1^ with an estimated 63 million people affected worldwide.^2^ In 2012 HF was responsible for an estimated health expenditure of $31 billion USD, a figure anticipated to see an increase of 127% by 2030.^3^ The increasing burden of HF on health care is primarily due to an aging population, as evidenced by the predominance of HF as a cause of hospitalization in individuals aged over 65 years.^4^ HF comprises an array of patients categorized by their symptoms and ejection fraction (EF), including those with reduced EF (EF<40%; HFrEF), midrange EF (EF between 40% and 49%), and preserved EF (EF>50%; HFpEF).^5^ Recent trends indicate that the prevalence of HFpEF is increasing relative to HFrEF, with estimates suggesting that 65% of patients with HF will have an EF>40% by 2020.^4^ Here, we review the current unmet needs in the management of HF and discuss how these needs may be addressed, focusing on the potential role of sodium‐glucose cotransporter‐2 (SGLT‐2) inhibitors.

## Substantial Unmet Needs in HF

### Urgent Need for Prevention of HF in Individuals at Risk, as Well as Early Recognition to Facilitate Treatment Before Hospitalization

The importance of prevention of symptomatic HF is highlighted in current international HF guidelines,^5^, ^6^ where the progressive nature of HF, from preclinical to clinical stages, is emphasized. Recognition of individuals at risk and the presence of preclinical cardiac structural/functional HF precursors are critical, because starting treatment at the preclinical stage may prevent HF progression and improve outcomes.^7^, ^8^, ^9^ Current US guidelines even recommend the use of biomarker screening to identify patients at increased risk of HF and, accordingly, adoption of preventative interventions.^6^

Even with symptomatic disease, major barriers to early diagnosis and treatment of HF remain, such as poor awareness of the disease among the general population^10^, ^11^, ^12^, ^13^ and suboptimal diagnosis by nonspecialist healthcare practitioners, who may have limited access to diagnostic tools such as echocardiography.^14^, ^15^ This is of particular concern in view of the variable clinical presentation of HF,^16^ as well as the overlap of symptoms (eg, shortness of breath, exercise intolerance) of the condition with common comorbidities, such as chronic obstructive pulmonary disease, chronic kidney disease (CKD), anemia, and diabetes mellitus.^17^, ^18^, ^19^ Delay in diagnosis of HF is associated with prolonged time to treatment and increased length of hospital stay and mortality.^20^, ^21^ As patient hospitalization is associated with an increased risk of mortality, early diagnosis is imperative.^22^ Studies indicate that patients hospitalized for HF have a 10% mortality rate at 30 days postdischarge^23^ and that the mortality rate at 1 year for patients admitted to a hospital is ≈20%.^24^ Furthermore, the readmission rate for HF at 6 months is 50%,^25^, ^26^ and the risk of mortality increases with each hospitalization.^13^

### Established Treatments for HFrEF Are Associated With Reduced Mortality

Although established treatments for HFrEF are associated with reduced mortality, these patients continue to have a poor prognosis, with many patients not receiving recommended doses of treatment. Neurohormonal antagonists (angiotensin‐converting enzyme [ACE] inhibitors/angiotensin receptor blockers [ARBs], β‐blockers, and mineralocorticoid receptor antagonists [MRA]) have been shown to reduce risk of mortality in patients with HFrEF.^27^, ^28^, ^29^ In addition, the PARADIGM‐HF (Prospective Comparison of ARNI [angiotensin receptor–neprilysin inhibitor] with ACEI [angiotensin‐converting‐enzyme inhibitor] to Determine Impact on Global Mortality and Morbidity in Heart Failure) trial showed benefit with ARNI, valsartan/sacubitril.^30^ Consequently, both European^5^ and US^6^ guidelines make the strongest recommendation for use of these neurohormonal agents, up‐titrated to optimal doses shown to be beneficial in clinical trials, to treat appropriate patients with HFrEF.

Despite these guideline‐recommended treatment options, mortality for patients with HFrEF remains high,^31^ with recent US registry data showing extremely poor 5‐year mortality (75%) and hospital readmission (82%) rates.^32^ A critical challenge in the management of HFrEF pertains to the large proportion of patients who do not receive guideline‐directed doses of treatment. A prospective European study found that of 2100 patients with HFrEF from 11 countries, only 22% were successfully up‐titrated over a 3‐month period to the recommended dose of ACE inhibitors/ARBs, and only 12% to the recommended dose of β‐blockers.^33^ The same study showed that patients with HFrEF treated with less than 50% of the recommended dose of ACE inhibitors/ARBs and β‐blockers had a greater risk of death and/or hospitalization for HF (hHF) than those reaching maximum dose.^33^ Moreover, in the prospective multinational ASIAN‐HF (Asian Sudden Cardiac Death in Heart Failure) registry, only 17% of patients received recommended doses of ACE inhibitors or ARBs, and 13% for β‐blockers.^34^ It is likely that failure to achieve recommended dosing is partly attributable to the length of time required to up‐titrate to maximum dose, with patients less likely to adhere to lengthy treatment processes without benefiting from immediate clinical improvements, as well as adverse events associated with current therapies (eg, hypotension, worsening of renal function, and hyperkalemia).

Considering the significant residual risk associated with HFrEF, there is an unmet need for disease‐modifying therapies that have an immediate impact on patient well‐being without dose‐limiting side effects. At the same time, optimizing treatment of HFrEF with existing therapies remains a key therapeutic goal. Initiatives that support implementation of best practice (eg, installation of in‐ and outpatient protocols within practices, ensuring that guidelines are readily available to all healthcare practitioners) may be valuable in facilitating appropriate management of HFrEF.

### No Therapy Has Been Shown to Convincingly Reduce Mortality in HFpEF

Several established treatments for HFrEF have shown no efficacy in trials of HFpEF, with no benefit demonstrated in the CHARM‐Preserved (Candesartan in Heart Failure: Assessment of Reduction in Mortality and Morbidity; candesartan),^35^ PEP‐CHF (Perindopril for Elderly People with Chronic Heart Failure; perindopril),^36^ I‐PRESERVE (Irbesartan in Patients with Heart Failure and Preserved Ejection Fraction; irbesartan),^37^ or TOPCAT (Treatment of Preserved Cardiac Function Heart Failure with an Aldosterone Antagonist; spironolactone) trials.^38^ Consequently, current European Society of Cardiology guidelines continue to state that “no treatment has yet been shown, convincingly, to reduce morbidity or mortality in patients with HFpEF,” with the only class I recommendations being diuretics for symptom control and treatment of comorbidities.^5^ The American College of Cardiology/American Heart Association guidelines similarly emphasize diuretics with a class I recommendation to relieve the symptoms of HF, with the only other class I recommendation being control of blood pressure. The American guidelines also recommend management of atrial fibrillation, addressing any myocardial ischemia with coronary revascularization, and use of other guideline‐recommended therapies for comorbid conditions. These guidelines also include a new class IIb recommendation for consideration of a mineralocorticoid receptor antagonist in appropriately selected patients with symptomatic HFpEF, particularly those with elevated natriuretic peptide levels, with close monitoring of potassium levels and renal function.^6^ This is based on a post hoc analysis of the TOPCAT trial, which showed efficacy of spironolactone in patients with HFpEF in the Americas but not in Russia/Georgia.^39^

### Cardiorenal Syndrome

**Figure 1:**
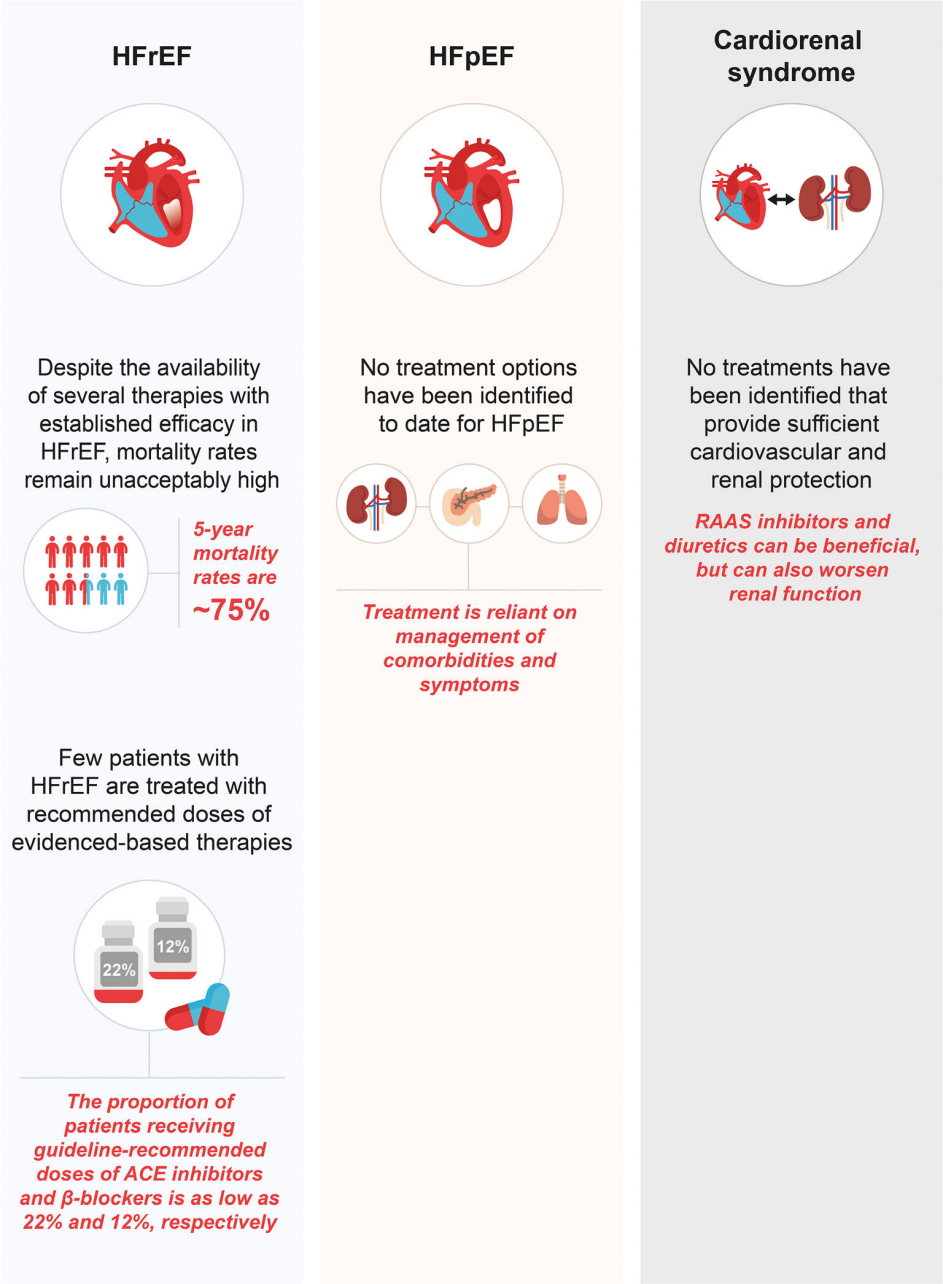
Key unmet needs in the management of HF.^5^, ^6^, ^32^, ^33^ ACE indicates angiotensin‐converting enzyme; HF, heart failure; HFpEF, heart failure with preserved ejection fraction; HFrEF, heart failure with reduced ejection fraction; RAAS, renin‐angiotensin‐aldosterone system.

The kidney and heart are inextricably linked, with acute or chronic disorder of 1 organ system capable of damaging the other; an interplay often referred to as cardiorenal syndrome.^40^ Studies have indicated that between 20% and 67% of patients with HF have CKD.^41^ Patients with both HF and renal insufficiency have ≈25% to 30% increased risk of mortality compared with patients with HF alone.^42^ The pathophysiology of renal disease in HF is complex: HF can lead to CKD through increased venous pressure and low cardiac output resulting in renal hypoperfusion, inflammation, and sympathetic activation. Conversely, kidney dysfunction may worsen HF through increased sodium and fluid retention, accelerated atherosclerosis, inflammation, anemia, uremic toxins, and neurohormonal activation.^43^ The progression of renal impairment in cardiorenal syndrome is often difficult to predict, making its management challenging. For instance, acute increases in serum creatinine and cystatin C of ≈0.3 mg/dL each may be prognostic for acute kidney injury and increased risk of hHF or death.^44^ However, transient worsening of renal function with appropriate neurohormonal blockade and/or diuresis, together with cardiovascular improvement, may not affect patient outcomes postdischarge.^44^ Most importantly, the presence of significant concomitant renal dysfunction severely limits the use of proven HF medications, such as ACE inhibitors, ARBs, MRAs, and ARNIs. In the absence of evidence‐based therapies capable of both renal and cardiovascular protection, outcomes for patients with cardiorenal syndrome may continue to be poor. Figure 1 summarizes the key unmet needs in HF.

## Emerging Evidence Suggests SGLT‐2 Inhibitors May Be Effective in Prevention of HF in Patients With Diabetes Mellitus

Diabetes mellitus is an independent risk factor for HF,^45^ with studies showing that subclinical atherosclerotic and nonatherosclerotic myocardial damage occurs early in the natural history of diabetes mellitus, often before diagnosis of the condition.^46^ SGLT‐2 inhibitors are approved for the management of type 2 diabetes mellitus (T2D) and have recently been investigated in several large, placebo‐controlled trials for cardiovascular safety as well as efficacy in patients with T2D.

### Cardiovascular Outcomes Trials of SGLT‐2 Inhibitors in Patients With T2D

EMPA‐REG OUTCOME (Empagliflozin Cardiovascular Outcome Event Trial in Type 2 Diabetes Mellitus Patients) was the first cardiovascular outcomes trial (CVOT) to investigate the effects of SGLT‐2 inhibition with empagliflozin on cardiovascular outcomes in T2D.^47^ In patients with T2D and established atherosclerotic disease (N=7020), empagliflozin met an exploratory end point of statistically significant reduction in hHF versus placebo.^47^ An absolute risk reduction (ARR) of 1.4% and relative risk reduction of 35% in hHF was observed in the empagliflozin group.^47^ Moreover, the cardiovascular benefits were shown to be independent of renal function^47^, ^48^ and glucose levels,^49^ as well as consistent when adjusted for baseline glycated hemoglobin (HbA_1c_) levels.^49^, ^50^

Building on results from the EMPA‐REG OUTCOME trial, the CANVAS study program (integrated data from 2 trials, CANVAS [Canagliflozin Cardiovascular Assessment Study] and CANVAS‐R [Canagliflozin Cardiovascular Assessment Study ‐ Renal]) investigated the SGLT‐2 inhibitor canagliflozin in patients with T2D and either established atherosclerotic disease (n=6656; 66%) or at high risk for cardiovascular events (n=3486; 34%).^51^ Canagliflozin significantly reduced the exploratory endpoint of hHF versus placebo (3.2% ARR; 33% relative risk reduction) in both patient subsets and also in patients with and without history of HF.^51^, ^52^

The DECLARE‐TIMI 58 (Dapagliflozin Effect on Cardiovascular Events‐Thrombolysis in Myocardial Infarction 58) trial, published in November 2018, is the first CVOT to include hHF or cardiovascular death as 1 of its primary end points. The DECLARE‐TIMI 58 trial investigated the effects of dapagliflozin versus placebo in a broad population of patients (N=17 160) with T2D who had either multiple cardiovascular risk factors (59.4%) or established atherosclerotic disease (40.6%).^53^ Dapagliflozin met 1 of its primary end points of a statistically significant reduction in hHF or cardiovascular death versus placebo, which was driven by a lower rate in hHF. Dapagliflozin was associated with an 0.8% ARR and 27% relative risk reduction in hHF. The benefit in hHF was consistent in patients with and without recognized atherosclerotic disease and also in patients with and without a previous history of HF.^53^ Recent subanalyses of the DECLARE‐TIMI 58 trial have shown that reductions in hHF with dapagliflozin also occurred both in patients with and without a previous MI and both in patients with and without peripheral artery disease, although those with previous MI and those with peripheral artery disease had a greater ARR.^54^, ^55^

A recent meta‐analysis of the three CVOT analyses showed that SGLT‐2 inhibitors, as a class, reduced the risk of hHF by 31%, and again that this risk reduction was consistent in patients with and without recognized atherosclerotic disease (≈30% reduction in risk of hHF in both subgroups) and in patients with and without a history of HF.^56^ Real‐world studies, such as CVD‐REAL (Comparative Effectiveness of Cardiovascular Outcomes in New Users of SGLT‐2 Inhibitors) and the ongoing EMPRISE (Non‐interventional Study on the Effectiveness and Safety of Empagliflozin Compared With DPP‐4 Inhibitors in Patients With Type 2 Diabetes in the United States) trial, have also demonstrated the positive effects of SGLT‐2 inhibitors on hHF prevention in patients with T2D irrespective of atherosclerotic disease status, providing consistent results in a practice‐based setting.^57^, ^58^, ^59^

### Safety of SGLT‐2 Inhibitors

Current evidence from trials of SGLT‐2 inhibitors in patients with T2D suggests these drugs are generally well tolerated. A common side effect of SGLT‐2 inhibitors is genital infections, which typically manifest early during treatment exposure.^47^, ^52^, ^53^, ^60^ Infections can be prevented if appropriate hygiene measures are taken, but should infection occur, it can be effectively managed.^60^ Diabetic ketoacidosis can occur in patients treated with SGLT‐2 inhibitors, although cases are very rare and mainly associated with the use of insulin.^47^, ^52^, ^53^, ^60^ Current guidance suggests that, should symptoms of diabetic ketoacidosis arise in patients receiving SGLT‐2 inhibitors, treatment should be discontinued immediately.^61^, ^62^

In the CANVAS trial, although the overall incidence of lower limb amputations was low, the frequency of these events was significantly greater in patients treated with canagliflozin versus placebo.^52^ Accordingly, European Medicines Agency guidance highlights the need for caution when prescribing SGLT‐2 inhibitors in patients at high risk of amputation.^63^ Whether the increased risk of amputation observed in the CANVAS trial is the result of a class effect across all SGLT‐2 inhibitors is the subject of significant debate. Notably, neither the EMPA‐REG OUTCOME nor DECLARE‐TIMI 58 trial showed an increase in the incidence of amputations with empagliflozin and dapagliflozin, respectively, compared with placebo.^47^, ^53^ Furthermore, a meta‐analysis of the results from the CANVAS, EMPA‐REG OUTCOME, and DECLARE‐TIMI 58 trials showed significant heterogeneity among the 3 trials with respect to amputations, suggesting that an increased risk of these events was evident only in the CANVAS trial.^56^ Conversely, recent registry data demonstrated an increased risk of amputation with SGLT‐2 inhibitors as compared with glucagon‐like peptide‐1 agonists.^64^ Although the study was confounded by performing analyses for SGLT‐2 inhibitors as a class, it is noteworthy that the study reports the use of canagliflozin to be rare, considering outcomes of the 3 major CVOTs concerning amputations.

Data are also inconsistent with regard to risk of bone fractures during SGLT‐2 inhibitor treatment. Although the incidence of fractures was significantly greater with canagliflozin versus placebo in the CANVAS trial, this finding was not mirrored in the EMPA‐REG OUTCOME, DECLARE‐TIMI 58, and CANVAS‐R trials^47^, ^52^, ^53^ or in registry data.^64^

Further research is clearly required to ascertain whether there is a genuine increased risk of amputations and fractures associated with SGLT‐2 inhibitor treatment and, if so, whether it is applicable to all drugs in that class or specific to an individual agent.

## Can SGLT‐2 Inhibitors Be Beneficial in the Treatment of HF?

Results from current CVOTs show promise for SGLT‐2 inhibitors in the prevention of HF in patients with T2D across a spectrum of cardiovascular risk. Whether SGLT‐2 inhibitors can be beneficial in the treatment of HF remains to be elucidated; efficacy in the prevention of HF does not necessarily translate to efficacy in the treatment of HF—the GISSI‐HF (GISSI Heart Failure) and CORONA (Controlled Rosuvastatin Multinational Trial in Heart Failure) trials exemplify this notion. Despite statins being the cornerstone pharmacological intervention in the prevention of heart disease, in both the GISSI‐HF and CORONA trials, rosuvastatin failed to show benefit in patients with chronic HF.^65^, ^66^

However, there is rationale to suggest that SGLT‐2 inhibitors may be beneficial in the treatment of established HF in patients with T2D. As previously discussed, a meta‐analysis of EMPA‐REG OUTCOME, CANVAS, and DECLARE‐TIMI 58 demonstrated cardiovascular benefit with SGLT‐2 inhibitors in patients with and without a history of HF.^56^ Moreover, subanalyses of the EMPA‐REG OUTCOME and CANVAS trials demonstrated that the ARR in hHF may be greater in patients with a history of HF than in those without; that is, at least for patients with high cardiovascular risk.^67^, ^68^ Only a minority of patients in the EMPA‐REG OUTCOME, CANVAS, and DECLARE‐TIMI 58 trials had investigator‐reported HF (≈10%), and no HF phenotyping (by EF or natriuretic peptide levels) was initially performed for these patients in the EMPA‐REG OUTCOME and CANVAS trials.^53^, ^68^, ^69^ The DECLARE‐TIMI 58 trial collected data on HF phenotype in patients with a history of HF (including history and etiology of HF, baseline EF, and functional class). Of the total 17 160 patients enrolled, 671 (4%) had an EF<45% and were classified as HFrEF; 1316 (8%) had a history of HF without a reduced EF (808 with recorded EF≥45% and 508 without a documented EF).^70^ Dapagliflozin reduced the primary composite end point of cardiovascular death or hHF to a greater extent in patients with HFrEF than in those without.^70^ This difference was driven by significant reductions in cardiovascular death in patients with HFrEF but not in those without; all‐cause mortality was also reduced in patients with HFrEF but not in those without. Reductions in hHF were seen irrespective of baseline EF. Of note, patients with reduced EF, in particular an EF<30%, derived greater relative cardiovascular benefit than those with a higher EF.^70^ It is also noteworthy that the cardiovascular benefits in patients with HFrEF were seen in patients previously treated with current evidence‐based therapies (eg, ACE inhibitors, β‐blockers, ARBs).^70^

A subanalysis of the CANVAS trial retrospectively assessed type of HF events in the study (HFrEF or HFpEF) using patients’ medical records data (reviewed by an original member of the Adjudication Committee who was blinded to patient treatment assignment). Of the 10 142 patients enrolled in the study, 101 had a first HF event considered as HFpEF (EF≥50%), 122 had a first event considered HFrEF (EF<50%), and 61 had a first event with unknown EF.^71^ The overall risk of HF events was shown to be reduced with canagliflozin versus placebo. The hazard ratio for HFrEF events was 0.69 (95% CI 0.48‐1.00), that for HFpEF events was 0.83 (95% CI 0.55‐1.25), and that for HF events with unknown EF was 0.54 (95% CI 0.32‐0.89). In the sensitivity analysis, where unknown EF events were assumed to be HFpEF, the updated hazard ratio for HFpEF events was 0.71 (95% CI 0.52‐0.97), and if the unknown EF events were assumed to be HFrEF events, the updated hazard ratio for HFrEF events was 0.64 (95% CI 0.48‐0.86). The authors, therefore, concluded that canagliflozin reduced the overall risk of HF events in patients with T2D and high cardiovascular risk, with no clear difference in effects on HFrEF versus HFpEF events.^71^ Unfortunately, EF at baseline was not available in this study. It will be interesting to see whether results from this subanalysis are reflected in ongoing dedicated HFrEF and HFpEF trials of SGLT‐2 inhibitors, particularly the latter in view of the lack of available treatment options for patients with HFpEF.

Accumulating mechanistic insights (Table) suggest that SGLT‐2 inhibitors may be valuable in the treatment of HF regardless of diabetes mellitus status and EF. SGLT‐2 inhibitors may exert cardioprotective effects through several distinct mechanisms, including (1) improvement in ventricular loading conditions secondary to reductions in preload (mediated by osmotic diuresis and natriuresis)^72^, ^73^ and afterload (potentially occurring via lowering of arterial pressure and stiffness);^74^, ^75^ (2) provision of an alternative cardiac energy supply in the form of cardiac ketones (specifically β‐hydroxybutyrate);^76^, ^77^ (3) direct inhibition of the sodium/hydrogen (Na^+^/H) exchanger in the myocardium,^78^ leading to reduction in or reversing of cardiac injury, hypertrophy, fibrosis, remodeling, and systolic dysfunction;^79^, ^80^, ^81^ (4) reduction in left ventricular mass and improvement in diastolic function^82^, ^83^ through inhibition of cardiac fibrosis (a feature of HF);^80^, ^84^, ^85^ (5) improvement in endothelial dysfunction;^86^, ^87^ and (6) stimulation of increased glucagon secretion,^88^, ^89^ potentially improving cardiac performance by either increasing cardiac index and fuel availability or decreasing peripheral vascular resistance.^90^

**Table 1:** Overview of Potential Mechanisms of Improved Cardiac Function With SGLT‐2 Inhibitors^72^, ^73^, ^74^, ^75^, ^76^, ^77^, ^78^, ^79^, ^80^, ^81^, ^82^, ^83^, ^84^, ^85^, ^86^, ^87^, ^88^, ^89^, ^90^, ^91^, ^92^, ^93^, ^94^

Potential Mechanisms
1. Stimulation of natriuresis
2. Stimulation of osmotic diuresis
3. Cardiomyocyte Na^+^/H exchanger inhibition
4. Increased myocardial energetics (via altered myocardial substrate metabolism)
5. Reduction in left ventricular mass
6. Improved systolic and diastolic function
7. Improved cardiac filling conditions secondary to reductions in preload and afterload
8. Increased circulating proangiogenic progenitor cells
9. Increased erythropoietin
10. Improved endothelial function
11. Reduction in myocardial CaM kinase II activity
12. Improved myocardial autophagy
13. Inhibition of cardiac fibrosis
14. Increased cardiac output, HR, O_2_ consumption, coronary blood flow mediated by increased levels of circulating glucagon

CaM indicates Ca^2+^/calmodulin‐dependent protein; HR, heart rate; SGLT‐2, sodium‐glucose cotransporter‐2.

### Renal Disease Adversely Impacts HF Outcomes, and SGLT‐2 Inhibitors May Offer Renal Protection

The recent CARMALINA trial highlights the deleterious effects of renal dysfunction on HF outcomes in patients with T2D, with or without prior history of HF. In the placebo group of the trial, there was a 2.7‐fold and a 4.2‐fold higher risk of hHF in patients with estimated glomerular filtration rate (eGFR) <60 mL/min per 1.73 m^2^ and <30 mL/min per 1.73 m^2^, respectively, compared with those with eGFR ≥60 mL/min per 1.73 m^2^.^95^ Moreover, in the SAVOR‐TIMI 53 (Saxagliptin Assessment of Vascular Outcomes Recorded in Patients With Diabetes Mellitus‐Thrombolysis in Myocardial Infarction 53) trial, worsening renal function was associated with a higher risk of both hHF and atherothrombotic events in patients with T2D during a median follow‐up of 2 years.^96^ Notably, with worsening renal function, risk of hHF was shown to be much higher than risk of atherothrombotic events. Whether improvement in renal function translates to cardiovascular benefit in patients with HF remains to be seen. However, it has been postulated that the kidney protection and natriuretic effects induced by SGLT‐2 inhibitors may account for the reductions in hHF in the EMPA‐REG OUTCOME, CANVAS, and DECLARE‐TIMI 58 trials. Moreover, the reduction in hHF in these trials was greater in patients with worse baseline renal function; a 40% reduction in hHF was observed in patients with eGFR <60 mL/min per 1.73 m^2^ compared with 31% and 12% reductions in patients with eGFR ≥60 to <90 mL/min per 1.73 m^2^ and eGFR ≥90 mL/min per 1.73 m^2^, respectively.^56^ Interestingly, although the magnitude of benefit from SGLT‐2 inhibition on hHF was greater (*P* value for interaction=0.0073) in patients with more severe renal disease at baseline, the benefit on progression of renal disease was lower in these patients (*P* value for interaction=0.0258). Most recently, results from the CREDENCE (Canagliflozin and Renal Events in Diabetes with Established Nephropathy Clinical Evaluation) trial, which investigated canagliflozin versus placebo on renal outcomes in 4401 patients with T2D and albuminuric CKD, support the notion that renal protection and cardiovascular benefit induced by SGLT‐2 inhibitors may be interlinked. The trial was prematurely stopped based on achievement of the prespecified efficacy criteria for the primary composite end point of time to first occurrence of end‐stage kidney disease, doubling of serum creatinine, or cardiovascular/renal death. Canagliflozin recipients also benefited from significant reductions in secondary end points of cardiovascular death or hHF and of hHF versus those on placebo. As in the DECLARE‐TIMI 58 trial, cardiovascular death was not reduced in the CREDENCE trial, suggesting that the reduction in the composite of cardiovascular death or hHF was driven by a reduction in hHF.^97^

### SGLT‐2 Inhibitors May Have Beneficial Effects in Patients Without Diabetes Mellitus

**Figure 2:**
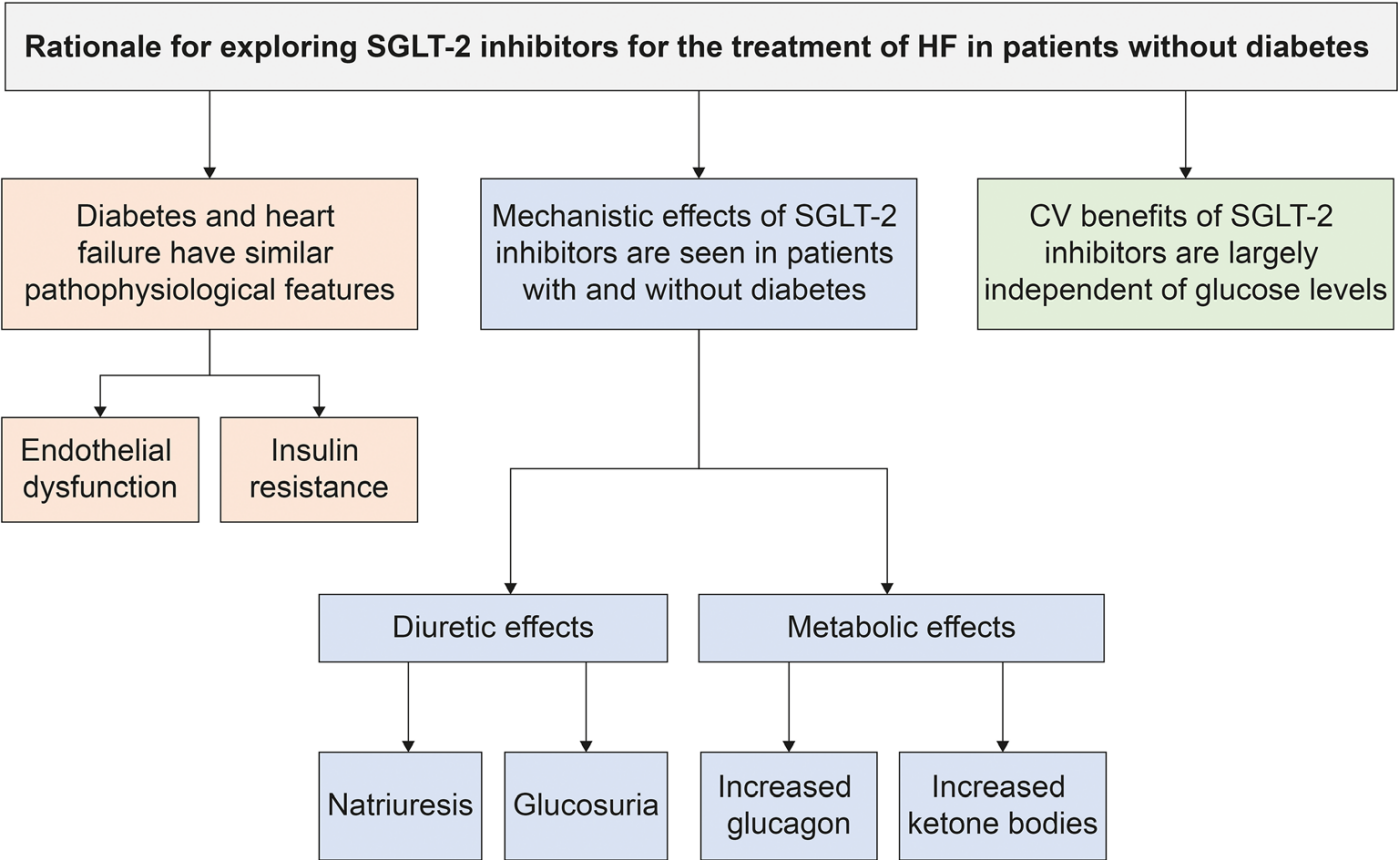
Mechanistic rationale for investigating SGLT‐2 inhibitors in HF beyond T2D. CV indicates cardiovascular; HF, heart failure; SGLT‐2, sodium‐glucose cotransporter‐2; T2D, type 2 diabetes mellitus.

Patients with HF, regardless of EF, have sodium and fluid retention as well as coronary, myocardial, and systemic endothelial dysfunction, even in the absence of overt diabetes mellitus. As the natriuretic (most notably), glucosuric, and metabolic effects of SGLT‐2 inhibitors have been demonstrated in patients with and without diabetes mellitus,^98^, ^99^, ^100^ it has been postulated that SGLT‐2 inhibitors may benefit patients with HF regardless of diabetes mellitus status (Figure 2). This has been demonstrated in several preclinical studies.^85^, ^101^, ^102^, ^103^ In a preclinical model of HF, empagliflozin treatment (or gene knockout simulation of SGLT‐2 inhibition) improved cardiac function.^101^ In preclinical models of MI, dapagliflozin has demonstrated attenuation of cardiac fibrosis, and empagliflozin has been shown to improve cardiac function and remodeling.^85^, ^102^ In other experimental models of HF without diabetes mellitus, empagliflozin prevented worsening of cardiac function.^103^

### Unanswered Questions and Future Direction

**Figure 3:**
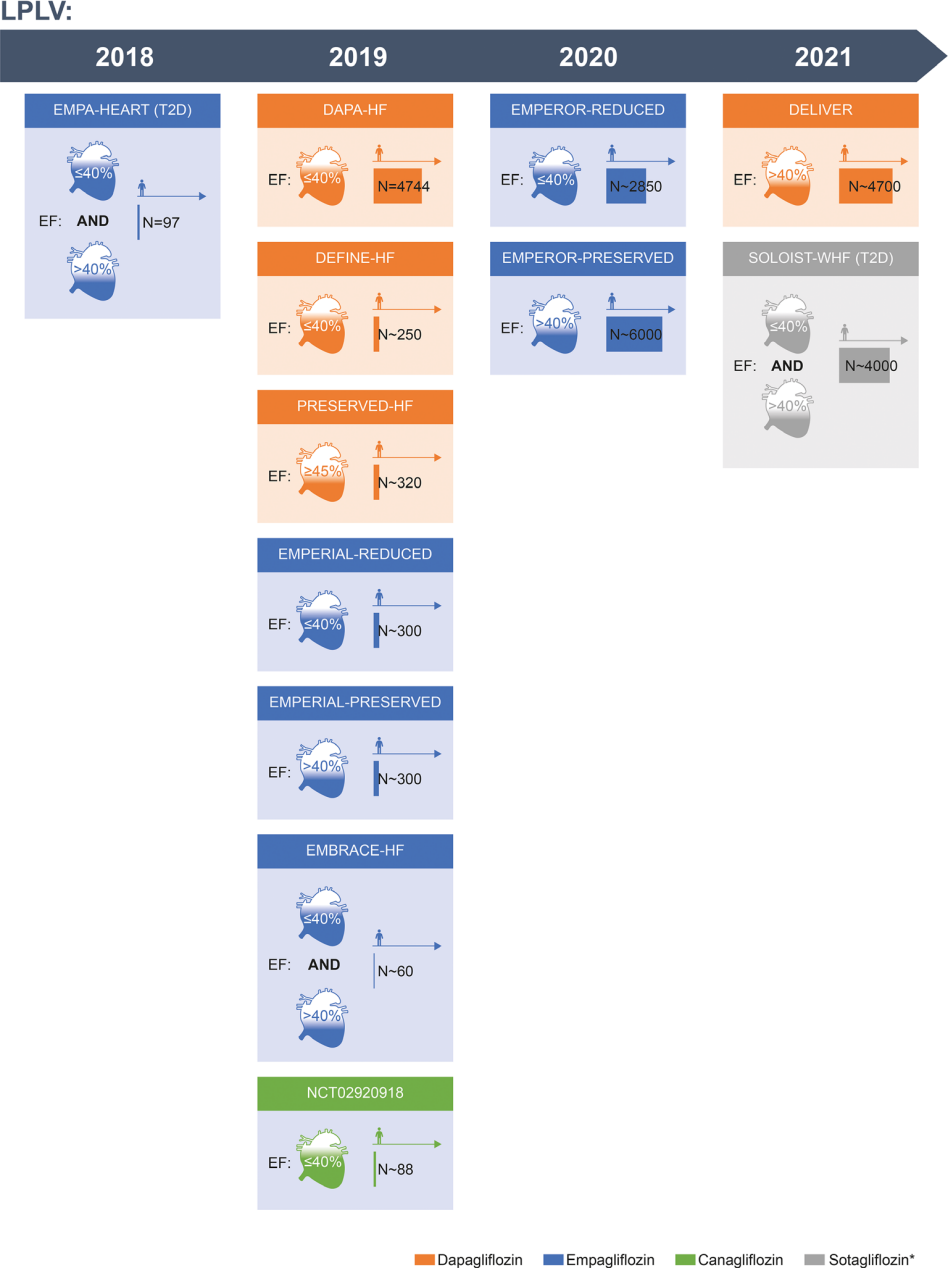
Ongoing trials of SGLT‐2 inhibitors in HF.^104^, ^105^, ^106^, ^107^, ^108^, ^109^, ^110^, ^111^, ^112^, ^113^, ^114^ *Dual SGLT‐1/SGLT‐2 receptor antagonist. EF indicates ejection fraction; LPLV, last patient last visit; SGLT‐2, sodium‐glucose co‐transporter‐2; T2D, type 2 diabetes mellitus.

Outcomes of several ongoing prospective studies of SGLT‐2 inhibitors in HF (Figure 3) are needed to fully evaluate the therapeutic potential of SGLT‐2 inhibitors in HF, with and without diabetes mellitus and with preserved or reduced EF. Of particular interest are the larger upcoming dapagliflozin and empagliflozin outcome trials (N>2000) in both HFrEF (DAPA‐HF [Dapagliflozin And Prevention of Adverse‐outcomes in Heart Failure] and EMPEROR‐Reduced [Empagliflozin Outcome Trial in Patients with Chronic Heart Failure with Reduced Ejection Fraction]) and HFpEF (DELIVER [Dapagliflozin Evaluation to Improve the Lives of Patients with Preserved Ejection Fraction Heart Failure] and EMPEROR‐Preserved [Empagliflozin Outcome Trial in Patients with Chronic Heart Failure with Preserved Ejection Fraction]), which are due to read out from 2019 onward (Figure 3) and may help establish whether there is a role for these SGLT‐2 inhibitors in HF independent of diabetes mellitus.

A recent randomized trial of empagliflozin versus placebo (EMPA‐HEART [Effects of Empagliflozin on Cardiac Structure, Function, and Circulating Biomarkers in Patients With Type 2 Diabetes]) in patients with T2D showed that empagliflozin treatment resulted in early and significant reduction in left ventricular mass regression, as detected by cardiac magnetic resonance imaging, which suggests reverse cardiac remodeling may be a possible contributor to the cardioprotective effects of SGLT‐2 inhibitors.^115^ By investigating the effects of SGLT‐2 inhibitors on HF‐specific biomarkers, hemodynamics, and cardiac structure/function, the PRESERVED‐HF (Effects of Dapagliflozin on Biomarkers, Symptoms and Functional Status in Patients with Preserved Ejection Fraction Heart Failure; primary end point: change from baseline in N‐terminal prohormone of brain natriuretic peptide [NTproBNP] at weeks 6 and 12 in patients with HFpEF), DEFINE‐HF (Dapagliflozin Effect on Symptoms and Biomarkers in Patients with Heart Failure; primary end point: change in NTproBNP at weeks 6 and 12 in patients with HFrEF), and EMBRACE‐HF (Empagliflozin Evaluation by Measuring Impact on Hemodynamics in Patients with Heart Failure; primary end point: change in pulmonary artery diastolic pressure from baseline to end of treatment with empagliflozin versus placebo) trials will help to further elucidate the potential beneficial effects of SGLT‐2 inhibitors on cardiovascular outcomes in patients with and without T2D. Sotagliflozin, currently being investigated in the SOLOIST‐WHF(Effect of Sotagliflozin on Cardiovascular Events in Patients with Type 2 Diabetes Post Worsening Heart Failure) trial, is a dual SGLT‐1/SGLT‐2 antagonist. Accordingly, the biology of this agent differs slightly from the 3 SGLT‐2 inhibitors prospectively studied to date. Thus, it is possible sotagliflozin may exhibit some drug‐specific effects, and it will be interesting to see whether the safety and efficacy impacts of this agent replicate those observed with SGLT‐2 inhibitors.

In addition to the recently published CREDENCE trial, several other trials dedicated to investigating renal and cardiovascular outcomes with SGLT inhibitors, which may shed light on the extent to which the renal effects of SGLT‐2 inhibitors contribute to the cardiovascular benefits these drugs appear to provide, are ongoing. These trials include, but are not restricted to, DAPA‐CKD (A Study to Evaluate the Effect of Dapagliflozin on Renal Outcomes and Cardiovascular Mortality in Patients with Chronic Kidney Disease; primary composite end point: time to first occurrence of ≥50% sustained decline in eGFR, reaching end‐stage renal disease or cardiovascular/renal death) and EMPA‐KIDNEY (A Multicentre International Randomized Parallel Group Double‐blind Placebo‐controlled Clinical Trial of Empagliflozin Once Daily to Assess Cardio‐renal Outcomes in Patients with Chronic Kidney Disease; primary composite end point: time to first occurrence of kidney disease progression [defined as end‐stage kidney disease, a sustained decline in eGFR to <10 mL/min per 1.73 m^2^, renal death, or a sustained decline of ≥40% in eGFR from randomization] or cardiovascular/renal death).

## Conclusion

HF is a highly debilitating condition affecting millions of individuals worldwide for which there remains substantial unmet needs for (1) prevention of HF by early recognition and treatment of individuals at risk; (2) improved adherence to treatment guidelines with respect to up‐titration of evidence‐based therapies among patients with established disease; and (3) additional effective and well‐tolerated therapeutic options, particularly in patients with HFpEF, as well as those with cardiorenal disease. SGLT‐2 inhibitors have emerged as a potential effective class of drug for the prevention of HF in patients with T2D. Mounting mechanistic evidence indicates that these drugs may also induce combined cardiac and renal beneficial effects and hold promise for the treatment of HF in patients with and without diabetes mellitus, as well as in both HFpEF and HFrEF. Whether this promise translates to clinical efficacy remains to be seen, but results of highly anticipated ongoing trials may help to provide the answer to this question.

## Sources of Funding

Funding for medical writing assistance was provided by AstraZeneca.

## Disclosures

Lam is supported by a Clinician Scientist Award from the National Medical Research Council of Singapore; has received research support from Boston Scientific, Bayer, Roche Diagnostics, AstraZeneca, Medtronic, and Vifor Pharma; has served as consultant or on the Advisory Board/Steering Committee/Executive Committee for Boston Scientific, Bayer, Roche Diagnostics, AstraZeneca, Medtronic, Vifor Pharma, Novartis, Amgen, Merck, Janssen Research & Development LLC, Menarini, Boehringer Ingelheim, Novo Nordisk, Abbott Diagnostics, Corvia, Stealth BioTherapeutics, JanaCare, Biofourmis, Darma, Applied Therapeutics, WebMD Global LLC, Radcliffe Group Ltd and Corpus. Subodh Verma is national coordinator for clinical trials sponsored by AstraZeneca, Boehringer Ingelheim, Sanofi, and Novonordisk; serves on the Study Executive Committee for clinical trials sponsored by Boehringer Ingelheim; is principal investigator for the NEWTON‐CABG, CAMRA‐1, ACE, and ENABLE‐CHIROPODY studies; and has received speaking and/or research support from Amgen, Abbott, AstraZeneca, Boehringer Ingelheim, Bayer, Bristol‐Myers Squibb, Merck, Janssen, Sanofi, Novartis, Lilly, and Novonordisk. The remaining authors have no disclosures to report.
